# Eating Disorders in Top Elite Beach Handball Players: Cross Sectional Study

**DOI:** 10.3390/children8030245

**Published:** 2021-03-22

**Authors:** Alejandro Martínez-Rodríguez, Manuel Vicente-Martínez, Javier Sánchez-Sánchez, Laura Miralles-Amorós, María Martínez-Olcina, Juan Antonio Sánchez-Sáez

**Affiliations:** 1 Department of Analytical Chemistry, Nutrition and Food Science, Faculty of Sciences, Alicante University, 03690 Alicante, Spain; amartinezrodriguez@ua.es (A.M.-R.); lma52@alu.ua.es (L.M.-A.); maria.martinezolcina@ua.es (M.M.-O.); 2 Alicante Institute for Health and Biomedical Research (ISABIAL Foundation), 03010 Alicante, Spain; 3 Faculty of Health Science, Miguel de Cervantes European University, 47012 Valladolid, Spain; mvmartinez11006@alumnos.uemc.es; 4 School of Sport Sciences, Universidad Europea de Madrid, 28670 Madrid, Spain; javier.sanchez2@universidadeuropea.es; 5 GDOT Research Group, Faculty of Sport, Universidad Católica de Murcia, 30107 Murcia, Spain

**Keywords:** eating disorders, Eating Attitudes Test-26, body weight, healthy habits, team sports

## Abstract

(1) Background: The preoccupation with the increasing appearance of eating disorders (ED) in athletes continues to grow, especially in athletes who practice team sports. ED severely affects the eating habits of the athletes, who tend to use unhealthy approaches to control their body weight. The development of nutritional education and early interventions by training staff is essential, and these factors are widely perceived as beneficial in sports medicine. This study evaluates the frequency at which beach handball (BH) players develop ED, also comparing the differences by sex and age (junior: adolescents vs. senior: young adults). In addition, the relation between body composition variables and ED was studied. (2) Methods: A descriptive and cross-sectional study was carried out in 69 top elite handball players (36 males and 33 females) from the Spanish National BH Team; who were separated by age (junior: adolescents and senior: young adults). The athletes completed the Eating Attitudes Test in its 26 item version (EAT-26). (3) Results: The prevalence of ED indicated that 11% of females had a high possibility of developing an ED, and 3% of males. Regarding the EAT-26 total score and subscales, no significant differences were found between female and male participants, or between the junior and senior categories. The correlations showed an association between body composition, in terms of body mass index, and the EAT-26 total score in both males and females. In the case of males, the correlation was negative. (4) Conclusions: Although there are no significant differences between sex or categories, it has been found that elite athletes are a population that is at high risk of developing ED.

## 1. Introduction

Eating disorders (ED) have a high probability of emerging in adolescence and adulthood rather than in childhood [1]. In this sense, ED are serious public health problems due to the increasing incidence in the population and the diversity of associated symptoms [2], even producing self-injuries [3]. Previous studies have found that participants with ED experience identity problems, feelings of hopelessness, a poor quality of life, and low psychological well-being. Moreover, suicidal behaviors and suicidal ideation have been found to be high in participants with ED [4]. They are also associated with a high risk of low bone mineral density and fractures [5]. High scores for eating-disorder behaviors in childhood or adolescence significantly predicted eating-disorder behaviors in young adulthood [6] causing an increased risk of later depressive symptoms.

ED are serious mental illnesses that are differentiated to others by the presence of disturbed eating behaviors combined with an altered perception of body composition or excessive concern about body image and weight. These disturbances lead to eating-related behaviors, such as vomiting, purging behaviors or extreme exercise, or restrictions. All of these actions negatively affect health, interpersonal relationships, mood, nutrition, and academic and work performance, as well as the quality of life of the affected individuals and their environment [7,8,9,10,11,12]. The classification of eating disorders can be found in multiple manuals, such as the Diagnostic and Statistical Manual of Mental Disorders (DSM-5) and the International Classification of Diseases (ICD-11) [13]. ED are subdivided into pica, rumination disorder, avoidant/restrictive food intake disorder, anorexia nervosa (AN), bulimia nervosa (BN), and binge-eating disorder (BED) [7].

ED commonly develop between late childhood and emerging adulthood, and are sometimes severe [14,15,16]. Mortality associated with ED is considerably high [13,17]. The prevalence of ED is 1.8%, increasing to 4.1% in the 14–20 age group [18]. Runfola et al. [19] observed a prevalence of 4.2% in men and women with a mean age of 21 (18–26 years). Other studies suggest that the earlier appearance of eating symptoms, the more severe health problems [20]. Marshall et al. [21] found no significant differences between junior and senior hockey players. Thus, there is a great deal of controversy regarding eating behaviors in relation to the early age of emergence [22]. In accordance with a review of 94 investigations, the mean prevalence of ED is 8.4% for females and 2.2% for males [13]. These data show ranges of prevalence from 3.3–18.6% and 0.8–6.5%, respectively [13].

Adolescents, women and high-income individuals are the groups at a higher risk of developing eating disorders [23]. The sex differences related to ED are possibly as high as 1 male for every 10 females [7]; also, notable research suggests that the prevalence in athletes is not that abundant [24,25,26]. Sundgot-Borgen and Klungland Torstveit (2004) found that the presence of eating disorders was significantly higher in female athletes (20.1%) than in male athletes (7.7%) [27].

Elite athletes who are also at risk are those who practice sports with weight categories or where physical fitness has a high influence on their success [28]. Different pressures such as personal, social, team and financial, which elite athletes have to face when competing, coupled with the high mental and physical demands presented by sports, make athletes more susceptible to ED [24]. Among these eating disturbances are many related behaviors such as severe food restriction, vomiting, extreme sweating or the use of diuretics and laxatives. Using these adverse behaviors to control weight is prevalent in the days leading up to a competition, and it also negatively influences the athlete’s performance at the event. Consequently, it is important to identify and prevent eating disorders in order to treat them as early as possible and obtain adequate results in both competition and health [29].

It has been shown that athletes are a population with a higher risk of presenting or developing eating disorder behavior than the general population [23]. Depending on the research, the prevalence around athletes ranges from 1 to 62%. This range is wide because it depends on sex and the type of sport studied. The sports observed to have the highest risk are distance running, wrestling and rhythmic gymnastics. This is the consequence of relating the performance improvement in these sports with being thin, because they are judged by their aesthetics or because they have weight categories [24,25,30,31].

However, this does not mean that the sports mentioned above are the only ones affected, participants in other sports are also at risk of suffering EDs, finding cases of eating disorders in team sports such as football, volleyball, handball or basketball [24]. Martinsen et al., (2013) observed no difference in the proportion classified as being “at risk” sports for ED between weight-sensitive and less weight-sensitive sports [32]. Other investigations show that in non-aesthetic sports with high competitive pressures, ED are more common than in weight-class sports [33]. Furthermore, a lot of controversial aspects have been found regarding the differences between individual sports and team sports [33].

An example that shows this controversy is presented by beach handball (BH) players, who have high nutritional and water requirements to be able to perform well in training and competition. These demands require the athlete to ingest large amounts of food, which means that they spend more time thinking about diet and sports performance, which can be counterproductive. Moreover, when athletes need a high physical involvement to practice their sport, it compromises mental health, often leading to severe symptoms of anxiety and depression. These situations are common comorbidities of ED.

The prevalence of athletes who reported severe or moderate dissatisfaction in ball sports such as handball is 20%, with 24.2% presenting mild dissatisfaction and 55.8% being categorized as having no dissatisfaction about their body [33]. These findings are relevant for performance sports, since it is undesirable for athletes seeking positive results and overcoming opponents to become affected by problems of emotional well-being, developing dissatisfaction with their body self-image and, subsequently, ED. According to the analysis, these ED are independent of the team sports practiced (e.g., volleyball, basketball, football or indoor handball), given that no association has been observed between modality and the presence of risk of ED and body self-image distortion [34].

As can be seen from the information explained above, elite BH players may be at risk of developing ED. However, no studies have been carried out in this population to date. Only one study has assessed the prevalence of ED in indoor handball players specifically, with results from the Eating Attitudes Test-26 (EAT-26) showing average scores of 9.92 ± 2.86 in male handball players and 11.55 ± 1.88 in female handball players, indicating a minimal risk of showing ED attitudes/behaviors [35]. However, these findings have not been replicated in elite BH players, who have a high body exposure and consequently a high body image pressure, which increases the risk of ED.

When there is continued exposure to sports culture and competition, it is possible that eating attitudes change and the risk of developing an ED increases with body maturation and puberty, which is related to body composition changes. Further research on the prevalence of ED in BH players is therefore needed to understand the risk to these athletes [36].

The general objective of the current investigation was to study the prevalence of a possible predisposition to ED in elite BH players. In addition, we aimed to compare the results between age groups (junior: adolescents and seniors: young adults) and sex (women vs. men), and identify the relationship between the different variables of the EAT-26 screening questionnaire and the variables of age, weight, height and BMI. The hypothesis was that female players would report higher scores than males on EAT-26, which indicates higher eating disorder symptomatology, and that there would also be associations between body composition in terms of body mass index and eating disorders.

## 2. Materials and Methods

### 2.1. Study Design

A descriptive and cross-sectional design was used to analyze the influence of sex and age on the unhealthy eating patterns in BH players. Furthermore, these data will assure better ED prevention among athletes, which could ultimately lead to greater health- and performance-related outcomes. The study was carried out with the best international players in this sport modality, thus representing the elite of BH players worldwide. All procedures were carried out in accordance with the Declaration of Helsinki (revision of Hong Kong in September 1989 and Edinburgh in 2000), and also in accordance with the recommendations for Good Clinical Practice of the EEC (document 111/3976/88 of July 1990). The University Human Research Ethics Committee of University of Alicante (Spain) granted approval to conduct this study (UA-2019-04-09).

### 2.2. Participants and Eligibility Criteria

A total of 69 BH players participated: 52.17% were males and 47.82% females, with a mean age of 20.9 ± 5.55 and 20.4 ± 5.18 years, respectively. All of the players that formed part of the current Spanish BH national teams, according to category and sex, participated. Exclusion criteria were the presence of chronic diseases, previously diagnosed mental illness, and refusal of informed consent. However, no athlete was excluded. All participants gave written informed consent before participating. In the present study, participants did not receive any compensation for their collaboration. In the case of participants who were underage, their parents or legal guardians gave the consent. Anonymity was preserved for all participants.

### 2.3. Data Collection

#### 2.3.1. Sociodemographic and Anthropometric Data

Questions regarding sex, age, and date of birth were asked to each of the BH players. Anthropometric data such as height and weight were measured using high quality, calibrated equipment. These measures were determined with light clothing and no shoes early in the morning, having not consumed any food or drink. Weight was measured in Kg and height in cm, and body mass index (BMI) was calculated (BMI = weight/size^2^, Kg/m^2^). The height of the athletes was determined using a mobile anthropometer (Seca 213, SECA Deutschland, Hamburg, Germany) to the nearest millimeter, and with the participant’s head maintained in the Frankfort Horizontal Plane position.

#### 2.3.2. Eating Disordered

Elite BH players completed the EAT-26 [37]. This is a commonly used [38,39,40] self-report questionnaire that determines whether an individual is at risk of developing an ED or not, using a 26-item 6-point Likert-type scale. A score of 20 or above was considered a cut-off point for identifying the possible presence of an ED. This test is not useful to diagnose, but rather to screen for ED and the need to see a mental health professional for a diagnosis according to the criteria of the Diagnostic Manuals (DSM-5 or ICD-11). EAT-26 is composed of 3 sub-scales: dieting (e.g., I avoid foods with sugar in them), bulimia and food preoccupation (e.g., I vomit after I have eaten) and oral control (e.g., I cut my food into small pieces) [37].

### 2.4. Statistical Analysis

For the data analysis, the programs Jamovi (Version 1.6.15, Sydney, Australia) and SPSS (version 16, Chicago, IL, USA) were used. First of all, the descriptive data (means and standard deviation) were calculated. For descriptive statistics (mean ± standard deviation) and inferential analysis, the Kolmogorov–Smirnov test was conducted to assess the normality of data followed by Levene’s test to determine the homogeneity (*p* > 0.05). Differences in BMI and EAT-26 scores between the different groups were analyzed using independent samples *t*-tests. The Student *t*-test was performed to compare sex differences in dieting. Analysis of covariance (ANCOVA) with the Bonferroni correction was used to compare differences between age groups (junior vs. senior), controlling the effect of BMI. Additionally, partial eta-squared (ηp^2^) was carried out with <0.25, 0.26–0.63, and >0.63 considering small, medium, and large effect sizes, respectively [41]. The correlations between BMI (kg/m^2^), and different scales of EAT-26 were determined using Pearson’s product-moment correlation coefficient (r), with 95% confidence intervals (CI).

## 3. Results

Sixty-nine (36 male; 33 female) top elite BH players aged from 15 to 35 (mean (M) = 20.6, standard deviation (SD) = 5.34 years) participated in this study; there were thirty-six male BH players, including 18 juniors (16.7 ± 0.46 years, 181 ± 5.9 cm, 78.1 ± 12.2 kg) and 18 seniors (25.0 ± 5.19 years, 188 ± 7.73 cm, 90.1 ± 13.4 kg), and thirty-three female BH players, with 18 juniors (16.7 ± 0.59 years, 167 ± 4.90 cm, 62.4 ± 7.29 kg) and 15 seniors (24.8 ± 4.71 years, 169 ± 5.31 cm, 64.9 ± 7.87 kg). Standard deviations and means were calculated for the principal variables, for male and female BH players separately (Table 1). The Student *t*-test showed no significant differences between female and male participants in EAT-26 scores and EAT-26 subscales.

Table 2 shows the mean and standard deviation values for BMI and EAT-26 subscales in female elite BH players. In total scores and subscale scores of EAT-26 (*p* > 0.05), there were no statistically significant differences between junior and senior female players. For male elite BH players, Table 3 shows the mean and standard deviation values for the BMI, total scores and EAT-26 subscales; there was also no significant difference in total scores and subscales of EAT-26.

**Figure 1:**
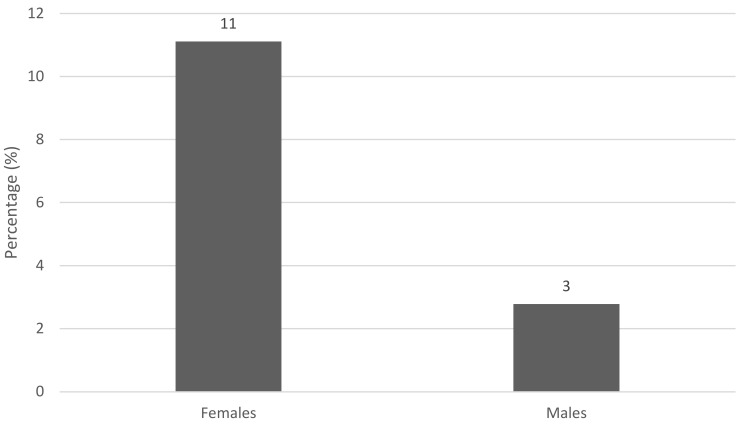
Percentage of players at risk to develop eating disorders.

However, as shown in Figure 1, in the case of female players, in both junior and senior teams, there were 2 players out of 18 who were at a high risk of developing an ED, representing 11% of the total number of females. For men, the rate was 3%, with only 1 player of the 36 who participated in the study being at risk.

In Table 4, statistically significant correlations (*p* < 0.05) are shown for the total sample, as well as for females and males individually between BMI, weight, height, age, EAT-26 total scores and EAT-26 subscale scores. Significant correlations between the basic body composition variables and the scores of the different EAT-26 scales were not observed. On the other hand, for females, the BMI and dieting subscale scores were positively correlated (r = 0.409; *p* = 0.018). Regarding weight, no statistically significant differences were observed, and in the dieting subscale, *p* = 0.073 was observed.

For males, statistically significant correlations (*p* < 0.05) between BMI, weight, height, age, EAT-26 total scores and EAT-26 subscale scores are shown. The total EAT-26 scores were positively associated with BMI (*p* = 0.028) and weight (*p* = 0.029). In addition, this correlation also exists with the oral control subscale score (*p* = 0.014 and *p* = 0.024, respectively).

## 4. Discussion

The purpose of this study was to identify the prevalence of a possible predisposition for elite BH players in developing an ED, by comparing age and sex differences and establishing possible correlations between the variables studied. BH is a recent sport, which means that it has received little intensive investigation to date, so available data are scarce. To the best of our knowledge, this is the first study to assess eating disorders in BH players. Only two studies have been published using the total punctuation of EAT-26 in multiple sports, including indoor handball [34,35]. In similar beach sports such as beach volleyball, the risk of ED occurrence has not been assessed.

The result of the evaluations showed that 14% (11% females and 3% males) of the BH players were prone to developing ED; this condition was not independent of nutritional status and body composition. These outcomes are similar to those reported by Kravchychyn et al. [34] and Perini et al. [42], who evaluated the presence of ED in athletes among the junior and senior categories, and found that 13.3% and 11.1% of those evaluated had a high risk, respectively. However, Kravchychyn et al. [34] found that the presence of ED has not been associated with nutritional status or body fat, and there has been no description in the literature of this relationship in athletes [34,35].

In contrast to the main hypothesis of this study, the results found no significant differences between male and female BH players on the EAT-26. However, in absolute values, there is a higher percentage in favor of elite female players. Studies have shown that females have more ED behaviors than males [43,44,45], often due to socio-cultural impositions and aesthetic patterns, making them a high risk group for the distortion of body self-image and ED [46]. This trend seems to be repeated in athletes [34]; furthermore, the results coincide with those of Fortes et al. [35], finding no differences in the EAT-26 between men and women indoor handball players [35].

As far as the EAT-26 score is concerned, no research has analyzed the subscales of this questionnaire in handball; for this reason, there is no scientific evidence to compare. Nevertheless, there has been research into the total score. Fortes et al. [35] obtained an EAT-26 total score in male junior handball players of 9.92 ± 2.86. The results of the present study were slightly lower, obtaining values of 7.11 ± 5.07 for males. For female junior players, Fortes et al. [35] obtained an EAT-26 total score of 11.55 ± 1.88. The score of the present study for female was lower, with values of 7.67 ± 7.69 being obtained. This difference may be due to the fact that the players in the Fortes et al. study were not professional players, and the scores are usually lower in elite players because the high level of competition can cause greater concern about weight and physical appearance in athletes [35]. Authors confirm that athletes with higher competitive levels are under greater pressure from coaches, sponsors, relatives, and friends, so these individuals appear to be a group of greater concern with morphological characteristics, since their goal is to obtain a better sporting performance [35].

In females, as can be seen in the correlations, there was a positive association between EAT-26 total score and BMI, in addition to the one between subscale dieting and BMI. Moreover, some studies found that overweight girls are more likely to present restrictive eating behaviors, worry about their weight and be less satisfied with their appearance [44,45]. On the other hand, in males, the correlations between BMI and total EAT-26 score and BMI and oral control subscale were negative. This makes sense because the high concern for muscle gain, the use of substances such as anabolic steroids, and homosexuality are sex-specific risk factors among males [30,47]. In addition, in the case of males, a strong preoccupation with gaining muscle mass has been found to be a major risk factor for ED and body dysmorphia [48]. This is confirmed by the results obtained in the present study, which show that there is a higher risk of developing an ED at lower BMI values.

The results found are relevant for performance sports, since it is undesirable for an athlete who is looking for positive results and overcoming adversaries to become involved with problems of emotional wellbeing, developing a dissatisfaction with their body self-image and, subsequently, with the aggravated picture of ED [34]. Among the most important aspects for the recovery from ED, are early detection and early medical, nutritional and psychiatric treatment [30,49,50]. In addition, when they are medically cleared, they need a close follow-up during training. Therefore, consensus recommendations emphasize the importance of multi-disciplinary treatment with an adjustment of both volume and type of exercise [49]. For this, it is also necessary the incorporation of nutritionists to the multidisciplinary team in beach handball.

Nevertheless, the current findings need to be considered alongside the possible limitations of the study. Therefore, as the current participants were elite BH players and the sample and size were not large, it is not recommended to generalize these results when studying other groups. Nevertheless, these results seem to provide essential data regarding BH and ED. Another limitation of this study is that only the EAT-26 questionnaire was used to analyze the risk of ED. For these reasons, future studies will have to use other measurement methods in addition to assessing high-risk behaviors with a clinical interview. Moreover, it would be of special interest to perform a longitudinal study, assessing the impact of nutritional education. It would be also relevant to study the different variables and risk factors of ED in children, adolescents and young adults.

All of this being said, the results from this manuscript and the comparison with previous works suggests that this study provides new information for the scientific field, specifically for research into psychological aspects of ED and factors that affect sports performance in BH.

Future research should focus on the origins and consequences of disordered eating behaviors in elite athletes. It would be interesting to analyze sport-specific risks, including the frequency of the assessment of weight, dieting pressure, the importance of weight loss, overtraining, early start of sport-specific training, injuries, impression motivation, personality traits and the impact of coaching partners in competition.

## 5. Conclusions

The results of the study indicate that there is a risk of ED in BH players, especially in females. Athletes tend to not seek support for adverse mental health situations because they perceive this action as a weakness (stigma, lack of understanding, poorer performance). Unhealthy eating habits are becoming a public health problem, especially in adolescent female athletes who tend to engage in harmful eating behaviors more often than males (strict diets, use of slimming products).

In order to make some progress in the prevention of ED, the identification and early treatment of mental health problems in elite athletes is a priority. In addition, investing in screening and nutritional education projects in all aspects of the athlete´s life is needed to promote a healthy lifestyle with appropriate eating behaviors. It is necessary the constant support of nutritionists in beach handball teams.

## Figures and Tables

**Table 1:** Beach-handball players descriptive and comparison (males vs. females).

	FEMALES (*n* = 33)			MALES (*n* = 36)			Comparison between Sexes	
	MEAN		SD	MEAN		SD	*t* Student	*p*
BMI	22.61	±	2.47	24.61	±	2.73	−3.188	0.002
EAT-26 total score	8.00	±	7.41	6.69	±	5.18	0.854	0.396
EAT-26 Dieting	4.09	±	4.38	3.56	±	3.79	0.544	0.588
EAT-26 Bulimia and Food Preoccupation	1.33	±	1.65	1.06	±	1.58	0.713	0.478
EAT-26 Oral Control	2.58	±	2.68	2.08	±	1.78	0.906	0.368

BMI = body mass index; SD = standard deviation; EAT-26, eating attitudes test-26; mean differences were significant at *p* < 0.05; df: degrees of freedom.

**Table 2:** Female beach handball (BH) players descriptive and comparison different EAT-26 subscales (junior vs. senior).

	JUNIOR (*n* = 18)			SENIOR (*n* = 15)			ANCOVA Comparison (Adjusting by BMI)				
	MEAN		SD	MEAN		SD	Mean Difference	df	*t*	*p*	np^2^
BMI	22.5		2.28	22.8		2.75					
EAT-26 total score	7.67	±	7.96	8.40	±	6.94	−0.487	30.0	−0.192	0.849	0.001
EAT-26 Dieting	3.72	±	4.28	4.53	±	4.60	−0.624	30.0	−0.433	0.668	0.005
EAT-26 Bulimia and Food Preoccupation	1.33	±	1.78	1.33	±	1.54	0.0148	30.0	0.0249	0.980	0.000
EAT-26 Oral Control	2.61	±	3.05	2.53	±	2.26	0.122	30.0	0.128	0.899	0.001

BMI = body mass index; SD = standard deviation; EAT-26 = eating attitudes test-26; mean differences were significant at *p* < 0.05; np^2^ = partial eta (effect size); *t* = *t* student; df: degrees of freedom.

**Table 3:** Male BH players descriptive and comparison different EAT-26 subscales (junior vs. senior).

	JUNIOR (*n* = 18)			SENIOR (*n* = 18)			ANCOVA Comparison (Adjusting by BMI)				
	MEAN		SD	MEAN		SD	Mean Difference	df	*t*	*p*	np^2^
BMI	23.9	±	2.82	25.4	±	2.50					
EAT-26 total score	7.11	±	5.07	6.28	±	5.39	−0.202	33.0	−0.118	0.907	0.000
EAT-26 Dieting	3.56	±	4.05	3.56	±	3.63	−0.501	33.0	−0.381	0.705	0.004
EAT-26 Bulimia and Food Preoccupation	1.33	±	1.68	0.778	±	1.48	0.419	33.0	0.762	0.451	0.017
EAT-26 Oral Control	2.22	±	1.99	1.94	±	1.59	−0.120	33.0	−0.206	0.838	0.001

BMI = body mass index; SD = standard deviation; EAT-26 = eating attitudes test-26; mean differences were significant at *p* < 0.05; np^2^ = partial eta (effect size); *t* = *t* student; df: degrees of freedom.

**Table 4:** Beach-handball players’ correlation between BMI and EAT-26 subscales.

	FEMALES				MALES				ALL			
	BMI	Weight	Height	Age	BMI	Weight	Height	Age	BMI	Weight	Height	Age
EAT-26 total score	0.317	0.200	−0.161	0.204	−0.366 *	−0.365 *	−0.218	0.028	−0.034	−0.156	−0.191	0.116
EAT-26 Dieting	0.409 *	0.316	−0.093	0.266	−0.227	−0.238	−0.153	0.089	0.053	−0.070	−0.128	0.172
EAT-26 Bulimia and Food Preoccupation	0.084	0.005	−0.129	0.183	−0.197	−0.203	−0.122	−0.087	−0.093	−0.152	−0.143	0.036
EAT-26 Oral Control	0.156	0.033	−0.212	0.016	−0.404 *	−0.375 *	−0.202	−0.031	−0.124	−0.204	−0.203	−0.009

BMI = body mass index; EAT-26, eating attitudes test-26; * = mean differences were significant at *p* < 0.05.

## Data Availability

The data presented in this study is available on request from the corresponding author. The data are not publicly available due to is personal health information.
